# Prognostic value of tumor regression grade (TRG) after oncological gastrectomy for gastric cancer

**DOI:** 10.1007/s00423-024-03388-8

**Published:** 2024-06-27

**Authors:** Francesco Abboretti, Styliani Mantziari, Laura Didisheim, Markus Schäfer, Hugo Teixeira Farinha

**Affiliations:** 1 Department of Visceral Surgery, Lausanne University Hospital, CHUV Rue du Bugnon 46, Lausanne, 1011 Switzerland; 2 Faculty of Biology and Medicine, University of Lausanne (UNIL), Lausanne, 1015 Switzerland

**Keywords:** Gastric cancer, Tumor regression grade, Gastrectomy, Neoadjuvant chemotherapy

## Abstract

**Purpose:**

Perioperative chemotherapy combined with surgical resection represent the gold standard in the treatment of locally advanced gastric cancer. The Mandard tumor regression score (TRG) is widely used to evaluate pathological response to neoadjuvant treatment. The aim of this study was to assess the prognostic value of TRG in terms of overall survival (OS) and disease-free (DFS).

**Methods:**

Retrospective analysis of all consecutive patients who underwent oncological gastrectomy after neoadjuvant chemotherapy from January 2007 to December 2019 for gastric adenocarcinoma was performed. Based on their TRG status they were categorized into two groups: good responders (TRG 1–2) and poor responders (TRG 3–5). Subsequent multivariable analyses were conducted.

**Results:**

Seventy-four patients were included, whereby 15 (20.3%) were TRG 1–2. Neoadjuvant regimens for TRG 1–2 vs. TRG 3–5 were similar: MAGIC (53% vs. 39%), FLOT (40% vs. 36%), FOLFOX (7% vs. 15%, *p* = 0.462). Histologic types according to Lauren classification for TRG 1–2 vs. TRG 3–5 were: 13% vs. 29% intestinal, 53% vs. 44% diffuse and 34% vs. 27% indeterminate (*p* = 0.326). TRG 1–2 group exhibited significantly less advanced ypT (46% vs. 10%, *p* = 0.001) and ypN stages (66% vs. 37%, *p* = 0.008), alongside a diminished recurrence rate (20% vs. 42%, *p* = 0.111). The 3-year DFS was significantly better in this group (81% vs. 47%, *p* = 0.041) whereas the disparity in three-year OS (92% vs. 55%, *p* = 0.054) did not attain statistical significance.

**Conclusions:**

TRG 1–2 was associated with less advanced ypT and ypN stage and better DFS compared to TRG 3–5 patients, without a significant impact on OS.

## Introduction

Gastric and esophagogastric junction (EGJ) adenocarcinoma remain a major cause of cancer-related death, while their incidence has been declining in the last years with several geographical variations [1]. Gastric cancer remains the 5th most common cancer in the world by incidence, accounting for 5.7% of all new cancer diagnoses and affecting over 1,000,000 patients worldwide annually, with a 5-year overall survival rate at 32% [2, 3]. In locally advanced, non-metastatic gastric and EGJ adenocarcinomas (≥ T2 or N+, M0), neoadjuvant chemotherapy followed by oncological gastrectomy has become the preferred therapeutic option [4–10]. Numerous trials extensively document the benefits of perioperative chemotherapy for locally advanced gastric cancer, primarily aiming at local downstaging and systemic disease control to mitigate the risk of distant metastasis [4–7]. For instance, findings from the FLOT4 trial demonstrated that perioperative FLOT regimen significantly enhanced overall survival compared to perioperative ECF/ECX, with a median overall survival of 50 months versus 35 months [5].

Beyond chemotherapy, other risk factors, including ethnicity [11], TNM stage [12–14], quality of surgical resection and lymphadenectomy [8, 15, 16], tumor localization [17, 18], histological type and genetic features [19, 20] impact on prognosis. Similarly, tumor response to preoperative treatment may influence oncologic outcomes [21]. Although various imaging techniques offer clinical evaluation such as computed tomography (CT), positron emission tomography (PET), endoscopic ultrasounds (EUS), the most reliable assessment hinges on histopathological analysis of the surgical specimen. Two classification systems are currently used for histopathological evaluation of tumoral response to treatment in gastric cancer: the first described by Mandard in 1994 [22] and the second by Becker in 2003 [23].

This retrospective study was aimed to investigate the prognostic value of the tumor regression grade (TRG) score according to Mandard in terms of probability of disease-free survival (DFS) and overall survival (OS) in a series of locally advanced gastric adenocarcinoma (GA) patients treated with curative intent.

## Materials and methods

This is a retrospective monocentric study including consecutive patients with resectable locally advanced gastric or EGJ (Siewert III) adenocarcinoma treated with neoadjuvant chemotherapy between January 2007 and December 2019 at the Lausanne University Hospital (CHUV), Switzerland. All types of oncologic gastrectomy (total and subtotal) were included, with a D2- lymphadenectomy (spleen and pancreas preserving) [16]. Indications for neoadjuvant chemotherapy at our institution adhere to the European Society for Medical Oncology (ESMO) guidelines, which recommend perioperative chemotherapy for patients with locally advanced resectable gastric cancer, specifically cT2 / cT3 or higher and/or N+ [9]. All neoadjuvant chemotherapy regimens were included; the most frequent types of chemotherapy used in our institution included EOX (*Epirubicin, Oxaliplatin and Capecitabine*), ECF (*Epirubicin, Cisplatin and continuous 5-Fluorouracil*) [4] and FLOT (*Fluorouracil, Leucovorin, Oxaliplatin and Docetaxel*) regimens [5, 6]. In order to compare baseline characteristics, clinico-pathological outcomes and long-term survival, patients were separated in two groups according to TRG: good (TRG 1–2) vs. poor (TRG 3–5) responders.

Exclusion criteria were patients who did not received pre-operative chemotherapy and underwent primary surgery, age < 18 and patient’s refusal to participate. Indications for gastrectomy were discussed in the institutional multidisciplinary tumor board.

The study was conducted according to the guidelines of the Declaration of Helsinki. Informed written consent was obtained for all participants and the study was approved by the Ethics Committee of Canton de Vaud (CER-VD, Lausanne, Switzerland: #2020 − 01114), with compliance to the Swiss policy of individual data protection.

### Data acquisition

Demographics, surgical and oncological data were retrieved from the prospectively maintained institutional database. The following variables were extracted: gender, age, ASA score, BMI, performance status according to WHO [24], histological type according to the WHO [25] and Lauren classification [26], TNM (8th edition TNM/IUCC staging system) [15], perioperative treatment regimen, surgical details, postoperative complications recorded according to the Clavien-Dindo classification [27], disease free survival (DFS) and overall survival (OS).

### Histopathological analysis

Pathologists with expertise in gastrointestinal oncology assessed all surgical specimens Regression score was evaluated for the primary tumor according to the Tumor Regression Grade (TRG) according to Mandard [22]. TRG 1 signifies a complete regression, with absence of residual cancer cells and fibrosis extending through the different layers of gastric wall; TRG 2 is characterized by the presence of rare residual cancer cells scattered through the fibrosis. While fibrosis continued to be dominant, TRG 3 is distinguished by an increase in the amount of residual cancer cells. TRG 4 shows residual cancer cells outgrowing fibrosis, whereas TRG 5 is associated with no histologic response to chemotherapy and absence of regressive changes. Patients with TRG grades 1–2 were considered as good responders, whereas those with TRG 3–5 as poor responders to chemotherapy.

### Statistical analysis

Continuous variables were presented as mean with standard deviation (SD) or median with interquartile range (IQR) according to their distribution. Categorical variables were reported as frequencies (%) and compared with chi-square test. Student’s t-test or Mann–Whitney test were used to compare continuous variables. Multivariate analyses were performed using Cox regression for Disease Free Survival (DSF), incorporating variables with univariate p-values ≤ 0.1. Kaplan–Meier survival curves were used to analyze time-to-event data and to compare survival between good and poor responders. All statistical tests were two-sided and a p-value of < 0.05 was used to indicate statistical significance. Statistical analyses were performed with GraphPad Prism 8 (GraphPad Software, Inc., La Jolla, CA, USA).

## Results

**Fig. 1:**
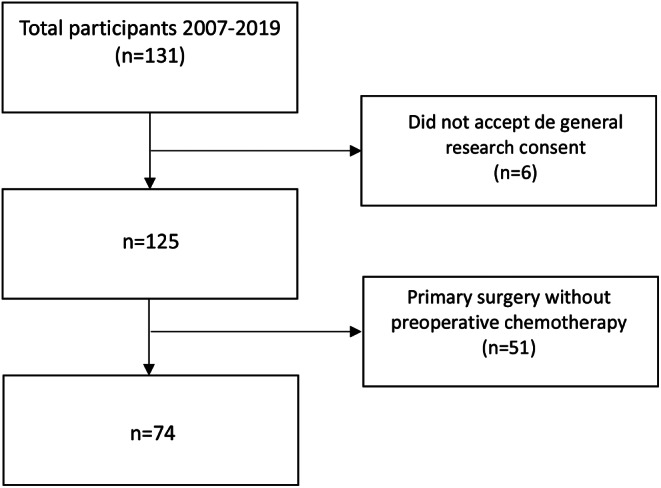
Inclusion flow-chart

One hundred thirty-one patients were screened for this study, 57 were excluded as described in Fig. 1. Finally, 74 patients (80% male) with a median age of 59 years (IQR: 51–71) were included. Median follow-up was 37 months (IQR: 14–60). Fifteen patients (20%) were considered good responders (TRG 1–2) and 59 (80%) poor responders. No differences were observed between the two groups regarding, age, gender, WHO performance status and neoadjuvant regimens. Baseline demographics are described in Table 1. Postoperative complications occurred in 38 patients (51%), 12 (16%) minor complications (Clavien I-II), and 26 (35%) major complications (Clavien IIIa-IV), with no statistical differences between the two groups (*p* = 0.590). There was no postoperative in-hospital mortality.

**Table 1:** Baseline Demographic and Clinical Characteristics of Patients Undergoing Oncological Gastrectomy for Gastric Cancer after Neoadjuvant Chemotherapy

	Overall *n* = 74	TRG 1–2 *n* = 15	TRG 3–5 *n* = 59	*p*-value
Gender Male Female	59 (80%) 15 (20%)	13 (86%) 2 (14%)	46 (78%) 13 (22%)	0.560
Age, median (IQR)	59 (51–71)	63 (55–67)	58 (50–71)	0.762
BMI (m/kg^2^), median (IQR)	25 (23–29)	25 (24–27)	25 (22–29)	0.662
ASA score I-II III	47 (64%) 27 (36%)	9 (60%) 6 (40%)	38 (64%) 21 (36%)	0.751
WHO perf. status 0 1–2	44 (60%) 30 (40%)	10 (66%) 5 (34%)	34 (58%) 25 (42%)	0.524
Gastric signet ring cell carcinoma (GSRC)	32 (32%)	3 (20%)	21 (36%)	0.249
Depth (cT) 2 3 4	12 (16%) 55 (74%) 7 (10%)	3 (20%) 11 (73%) 1 (7%)	9 (15%) 44 (75%) 6 (10%)	0.692
Node (cN) 0 1 2 x	20 (27%) 51 (69%) 2 (3%) 1 (1%)	5 (33%) 8 (53%) 1 (7%) 1 (7%)	15 (25%) 43 (73%) 1 (2%) -	0.423
Neoadjuvant regimen MAGIC (EOX, CF) Platine, 5-FU FLOT FOLFOX other	31 (42%) 3 (4%) 27 (36%) 10 (14%) 3	8 (53%) - 6 (40%) 1 -	23 (39%) 3 (5%) 21 (36%) 9 (15%) 3	0.462
Surgical details Subtotal gastrectomy Total gastrectomy	17 (23%) 57 (77%)	2 (13%) 13 (87%)	15 (25%) 44 (75%)	0.327
Surgical approach Open Laparoscopic	52 (70%) 22 (30%)	11 (73%) 4 (27%)	41 (69%) 18 (31%)	0.775
Lymph node dissection Spleen preserving D2 Classic D2	67 (91%) 7 (9%)	13 (87%) 2 (13%)	54 (92%) 5 (8%)	0.624
TRG breakdown TRG 1 TRG 2 TRG 3 TRG 4 TRG 5	7 (9%) 8 (11%) 17 (23%) 34 (46%) 8 (11%)	7 (46%) 8 (54%) - - -	- - 17 (29%) 34 (58%) 8 (13%)	-

Legend: Data are presented as the number of patients (percentages). IQR: interquartile range, BMI: Body Mass Index, ASA: American Society of Anesthesiologists, WHO: World Health Organization, SRC: Signet Ring Cell, EOX: Epirubicin, Oxaliplatin and Capecitabine, ECF: Epirubicin, Cisplatin and continuous 5-Fluorouracil, 5-FU: 5-Fluorouracil, FLOT: Fluorouracil, Leucovorin, Oxaliplatin and Docetaxel, FOLFOX: Leucovorin, 5-Fluorouracil, Oxaliplatin

### Histopathological characteristics

Significant differences were observed between the two groups in terms of tumoral staging, as shown in Table 2: ypT staging was lower in TRG 1–2 group (*p* = 0.001) and, similarly, a higher rate of ypN0 staging was found in the good responder group (66% vs. 22%, *p* = 0.008). Complete resection margins (R0) were achieved in 81% of patients, with a 100% R0 resection in the pathological responder group (TRG 1–2) and 76% in the TRG 3–5 group (*p* = 0.001). No differences were found in terms of tumor type according to the WHO and Lauren classifications.

**Table 2:** Histopathological features of patients undergoing oncological gastrectomy for gastric cancer after neoadjuvant chemotherapy

	Overall *n* = 74	TRG 1–2 *n* = 15	TRG 3–5 *n* = 59	*p*-value
Depth (pT)				0.001
0	5 (7%)	5 (33%)	-	
1a, 1b	8 (11%)	2 (13%)	6 (10%)	
2	9 (12%)	2 (13%)	7 (12%)	
3	35 (47%)	5 (33%)	30 (51%)	
4a, 4b	17 (23%)	1 (8%)	16 (27%)	
Node (pN)				0.008
0	32 (43%)	10 (66%)	22 (37%)	
1	12 (16%)	3 (20%)	9 (15%)	
2	10 (14%)	2 (14%)	8 (14%)	
3	19 (27%)	-	19 (34%)	
x	1	-	1	
Metastases (pM)				0.033
0	70 (95%)	15 (100%)	55 (93%)	
1	4 (5%)	-	4 (7%)	
Grading				0.994
G1	5 (7%)	1 (7%)	4 (7%)	
G2	20 (27%)	5 (33%)	15 (25%)	
G3	46 (62%)	7 (47%)	39 (66%)	
undetermined	3 (4%)	2 (13%)	1 (2%)	
Resection margins				0.001
R0	60 (81%)	15 (100%)	45 (76%)	
R1	14 (19%)	-	14 (24%)	
WHO classification				0.539
Mucinous	9 (12%)	1 (7%)	8 (14%)	
Tubular	25 (34%)	6 (40%)	19 (32%)	
SRC	21 (28%)	2 (13%)	19 (32%)	
Mixed	3 (4%)	2 (13%)	1 (2%)	
other	16 (22%)	4 (27%)	12 (20%)	
Lauren classification				0.326
Intestinal	19 (26%)	2 (13%)	17 (29%)	
Diffuse	34 (46%)	8 (53%)	26 (44%)	
Indeterminate	21 (28%)	5 (34%)	16 (27%)	

Legend: Data are presented as the number of patients (percentages). WHO: World Health Organization, SRC: signet ring cell carcinoma or poorly cohesive carcinoma

### Recurrence and survival

**Fig. 2:**
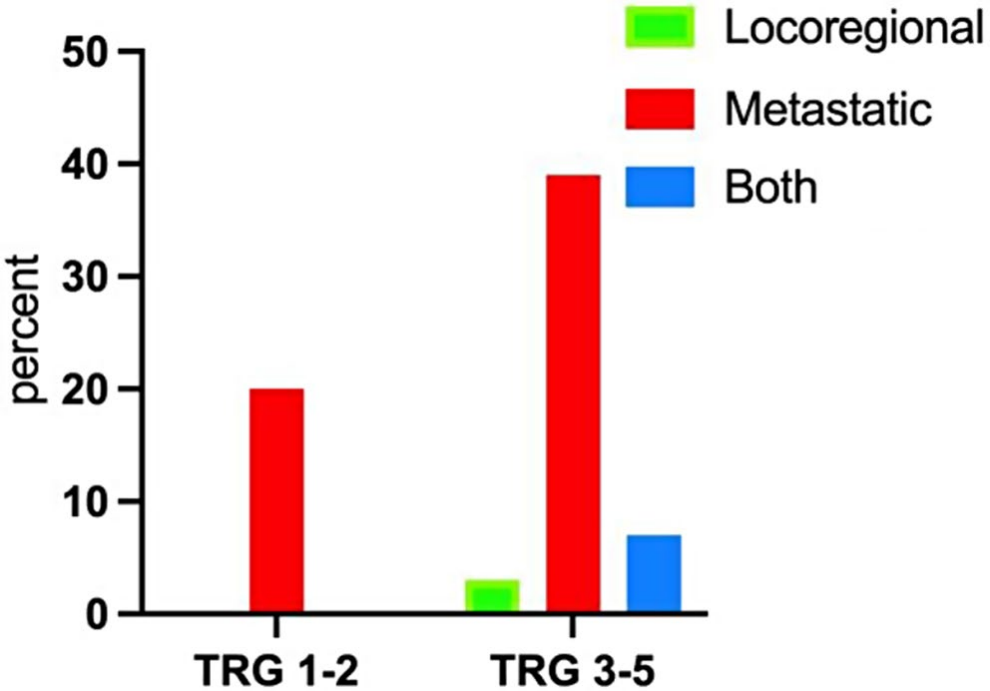
Recurrence rate in percent according to TRG 1–2 versus TRG 3–5

**Fig. 3:**
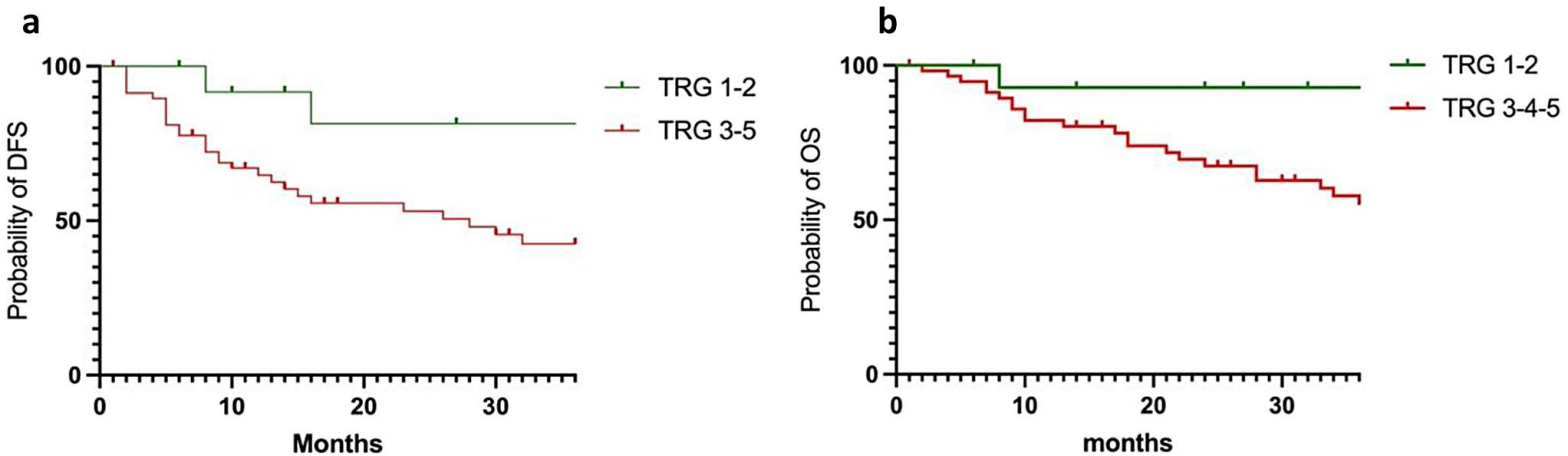
Kaplan Meyer survival curves for disease free survival (DSF, *p* = 0.041) (**a**) and overall survival (OS, *p* = 0.054) (**b**) at 36 months

TRG 1–2 patients had similar recurrence rates compared to TRG 3–5 (*n* = 3, 20% versus *n* = 25, 42% respectively, *p* = 0.111). No locoregional recurrence was observed in TRG 1–2 group as showed in Fig. 2. Patients in TRG 3–5 group had a significant higher metastatic risk (46% vs. 20%, *p* = 0.001). Improved DFS was observed at 36 months for TRG 1–2 patients compared to TRG 3–5 (81% vs. 47%, *p* = 0.041). No significant difference was observed in 3-year OS (92% vs. 55%, *p* = 0.054) (Fig. 3). TRG was not found to be an independent predictive factor of DFS after multivariable analysis (*p* = 0.135), unlike nodal invasion (pN) (*p* = 0.196) and resection margins (R0) (*p* = 0.524). Moreover, pT stage was found to be an independent predictive factor (*p* = 0.024) (Table 3).

**Table 3:** Cox regression analysis for Disease Free Survival (DFS)

	HR	95% CI for OR	*p*-value
TRG	0.813	[ -0.179 to 1.989]	0.135
pT stage	0.692	[ 0.112 to 1.322]	0.024
nodal invasion (pN)	0.182	[-0.109 to 0.437]	0.196
resection margins (R0)	-0.251	[-1.039 to 0.524]	0.524

After univariate analysis, variables with p-values ≤ 0.1 were incorporated into a multivariate analysis using Cox regression

Legend: *CI: confidence interval, HR: hazard ratios*

## Discussion

In this study, although TRG 1–2 was associated with improved disease-free survival compared to TRG 3–5, its role as an independent prognostic factor was not established in the multivariable analysis. This suggests that the ability of TRG to serve as a standalone predictor of patient outcomes, independent of other clinical and pathological variables, is uncertain. The lack of significant findings may be attributed to the influence of additional variables considered in the analysis and the limited sample size of the study population.

As described by Al-Batran et al. [5], only a minority of patients show a good pathological response after neoadjuvant chemotherapy. In our series, 20.3% of patients presented a TRG 1–2, which is consistent with previously published data, reporting 17 − 28.6% TRG 1–2 rates for gastric adenocarcinoma [28–31]. The correlation between the Lauren histological type and TRG is still not clear. Some studies [30, 32] found a negative impact of the diffuse type on tumor regression. In this study, we were unable to demonstrate a correlation between Lauren classification and histopathological tumor response after neoadjuvant chemotherapy probably due to the small sample size considered.

Moreover, TRG was not associated with the presence signet ring cells (SRC) in our study. SRC histology has been reported in up to 25% of all gastric adenocarcinomas in the US [20]. They are usually associated with poor survival outcomes due to the high risk of submucosal infiltration and distant micro-metastases. Although signet ring cell adenocarcinomas have been suggested to have worse histopathological response to treatment [33]. Notwithstanding, several studies did not find any correlation between SRC adenocarcinoma and poor histopathological response [29, 31, 34]. Xie et al. data reveal that the oxaliplatin-based regimen failed to improve OS and DFS in patients with signet ring cell adenocarcinoma, indicating that the oxaliplatin-based regimen may not be the optimal choice of neoadjuvant chemotherapy for these patients [34].

One of the most important aims of the neoadjuvant chemotherapy is to provide local downstaging and higher R0 resection rate to obtain a better survival. Evaluation of histopathological response is mandatory to assess the tumor regression grade following the perioperative treatment. Previous studies have noted the importance of histological evaluation of lymph nodes metastasis and pathological response to chemotherapy on the surgical specimen as predictors of survival after chemotherapy plus resection, especially in the MAGIC trial [35]. This study shows the correlation between TRG, ypTNM and resection margins. These results reflect those of Derieux et al.) [36] and Lombardi et al.) [31], who also found that TRG 1–2 patients had a lower ypTN score compared to non-responders. The present study shows also a higher R0 resection in good pathological responders, as well as lower ypT and ypN scores in TRG 1–2 patients. Nevertheless, in this study we did not compare the preoperative clinical staging score (cTNM) with the postoperative post-neoadjuvant treatment staging score (ypTNM). Furthermore, due to the limited number of participants in our study and the wide range of neoadjuvant regimens, no association between preoperative chemotherapy and TRG was found.

A multicentric study has demonstrated how TRG evaluation was a good prognostic predictor for advanced gastric cancer patients receiving neoadjuvant chemotherapy [34]. This study, including 249 patients, showed a significantly better survival outcomes for good histopathological responders. At multivariate survival analysis it was demonstrated that TRG was an independent prognostic factor of poorer OS. These findings were strongly supported by the results of a meta-analysis published by Tomasello et al. in 2017, based on 17 studies Gathering primarily studies on esophageal and junctional tumors [21]. The authors concluded that good pathological response was significantly correlated with an improvement in overall survival.

This study provides insights into the correlation between good histopathological response and survival outcomes. Moreover, our data suggests that TRG may predict the risk of developing distant metastasis. TRG classification could represent a potential predictor of better survival outcomes in case of good pathological response to neoadjuvant chemotherapy. Wang et al., demonstrate enhanced OS outcomes in patients exhibiting favorable pathological responses, while our study found a correlation between better DFS and TRG 1–2 [37]. Several questions remain to be answered, especially the potential benefit of changing the adjuvant chemotherapy regimen in pathological non-responders (TRG 3–5), following the idea that post-operative treatment should be adapted to histopathological tumor characteristics.

TRG determination serves as a logical complement to traditional pathologic TNM staging. By defining the quantity of residual cancer cells after neoadjuvant therapy, further investigations are necessary to elucidate the quality of neoplastic cells and their behavior, particularly in patients without lymph node metastasis. Identifying factors indicative of a more aggressive phenotype and/or resistance to chemoradiotherapy may enhance the prognostic value of TRG and enable treatment tailoring.

The current study has certain limitations, which are mostly related to its retrospective nature and the small cohort of patients. Small differences between the groups might have passed undetected due to type II error. Furthermore, we need to consider the use of different types of chemotherapy regimens among the years and the inclusion of gastric and esophago-gastric junction adenocarcinoma (Siewert III), which may present several biological differences. A notable limitation of this study is the postoperative discovery of metastatic disease in some patients, which were identified as pM1 during the final histopathological analysis. Although all surgeries were conducted with curative intent under the assumption of no metastatic spread (cM0) based on preoperative evaluations, the unexpected findings of metastases such as peritoneal carcinomatosis at the time of surgical resection highlight a potential confounder in assessing the true prognostic impact of TRG. Another limitation is the absence of data on cancer localization and Borrmann classification. Their absence limits our ability to fully assess the impact of tumor location and morphology on treatment efficacy and tumor regression.

## Conclusions

Tumor regression grade evaluation should be done systematically during the histopathological analysis. This European cohort study shows a correlation between good pathological responders to pre-operative chemotherapy (TRG 1–2) and disease-free survival. More research is needed to confirm the suitability of changing the postoperative chemotherapy regimen in poor responders (TRG 3–5).

## Data Availability

No datasets were generated or analysed during the current study.
